# Detection and Identification of Pathogenic *Leptospira* spp. Serogroups in Europe between 2017 and 2020 Applying a Novel Gene-Based Molecular Approach

**DOI:** 10.1155/2024/1101841

**Published:** 2024-06-11

**Authors:** Jasmin Wenderlein, Theresa Zitzl, Nathalie Dufay-Simon, Nathalie Cachet, Nikola Pantchev, Marine Le Guyader, Célia Fontana, Natalia Bomchil, Jean-Philippe Tronel, Lionel Cupillard, Reinhard K. Straubinger

**Affiliations:** ^1^ Chair for Bacteriology and Mycology Institute for Infectious Diseases and Zoonoses Department of Veterinary Sciences Faculty of Veterinary Medicine LMU Munich Sonnenstraße 24, Oberschleißheim85764Germany; ^2^ Boehringer Ingelheim Animal Health Global Innovation 813 Cours du Troisieme Millenaire, Garnier, Saint-Priest69800France; ^3^ IDEXX Laboratories GmbH Humboldstraße 2, Kornwestheim70806Germany; ^4^ USC 1233 RS2GP VetAgroSup INRAE Université de Lyon 1 Avenue Bourgelat, Marcy L'étoile, Lyon69280France; ^5^ EVOTEC ID Lyon (SAS) 40 Avenue Tony Garnier, Lyon69007France; ^6^ LyonBiopole 63 Avenue Tony Garnier, Lyon69007France

## Abstract

Leptospirosis is a neglected but reemerging worldwide zoonotic disease. Due to a rise in global temperature, precipitation, and urbanization, the risk of acquiring an infection with *Leptospira* (*L*.) spp. increases in Europe. One species affected by leptospirosis living close to the human is the dog. Even though dogs can shed low numbers of leptospires, their value as sentinel animal aiding in understanding environmental reservoirs might be even more important for human and animal health. Therefore, it is crucial to study the prevalence of pathogenic *L*. spp. in dogs and tailor coordinated protective measures like vaccination. This study aimed to screen a collection of purified DNA extracted from canine field samples (blood and urine, *n* = 239) collected in Europe from dogs suspected of leptospirosis and found positive by the *lipL32* PCR used for diagnostics. A new simple and effective molecular approach was developed to identify different leptospiral serogroups using first a 16S rRNA PCR and 16S rRNA gene sequencing for genomospecies determination followed by a PCR targeting the *rfb* gene locus responsible for LPS biosynthesis for serogroup classification. In total, 172 DNA samples were successfully tested. Results show that *L*. Icterohaemorrhagiae was detected as the most prevalent serogroup in Europe (53%), followed by *L*. Australis serogroup (13%). At lower percentages, *L*. Pomona (5%), *L*. Autumnalis (4%), and *L*. Sejroe (2%) were identified. This work emphasizes that current L4 vaccines are relevant and should confer a high efficacy profile at least against *L*. Icterohaemorrhagiae and *L*. Australis serogroups.

## 1. Introduction

The infection with *Leptospira* (*L*.) spp. organisms, causing leptospirosis in susceptible hosts, is considered a reemerging bacterial zoonotic disease, and especially in Europe, this pathogen has been neglected. Infection with *L*. spp. occurs either through direct contact with an infected individual or through water or soil contaminated with urine from infected animals [1]. With most cases occurring in tropical or subtropical and rural areas [2], we in Europe might think ourselves relatively safe from acquiring this infection. Associations between leptospirosis and open sewers, high annual rainfalls, and a warm humid climate have been published for tropical regions [3]. Heavy precipitation and floods can transport animal pathogens such as *L*. spp. from pastures into waterways and thus result in waterborne outbreaks [4, 5, 6], especially when work or free time activities are conducted in muddy environments [7, 8]. Additionally, urbanization and the resulting reduction in biodiversity allow certain rodents like *Rattus rattus*, a main transmitter of *L*. Icterohaemorrhagiae organisms, to assemble in cities [9]. These risk factors reflect the situation in Europe when considering climate change with its increase in temperature and extreme weather events like heavy rainfall and flooding [4, 5, 6]. When combining the risks of climate change, heavy rainfalls, and urbanization an increase in infections with *L*. spp. in Europe might be expected [10]. Besides humans, various mammals can harbor *L*. spp. in their renal tubules [11], additionally, many of these animals can serve as reservoir hosts [12, 13, 14, 15]. Mammals living in close relationships with humans and sensible to infections with *L*. spp. are farm and companion animals [1]. Leptospirosis in companion animals like dogs and cats is considered a One-Health concern [1], especially as animals may not necessarily display clinical signs and can shed *L*. spp. with the urine [16]. While leptospirosis in cats is reviewed elsewhere [16, 17], this work focuses on leptospirosis in dogs. Canine leptospirosis in some cases has been described to occur without clinical signs, while in some cases, a wide spectrum of clinical signs can be observed [1]. These include lethargy, anorexia, and fever, while the involvement of kidneys, lungs, liver, and the vascular system has been described in descending quantity [18, 19]. In general, due to unspecific clinical signs, the identification of dogs infected with *L*. spp. and the initiation of diagnostic measures and consequently the application of therapeutics remains complicated. The diagnostic confirmation of canine leptospirosis is either conducted by detecting antibodies in the microscopic agglutination test (MAT) [20, 21], more frequently since several years by detecting leptospiral DNA in blood or urine using specific PCR methods [22], or rarely by culturing viable organisms in specific media over prolonged periods [1]. However, all listed tests come with limitations. The MAT is known for low and variable sensitivity, specificity, and repeatability [23]. Furthermore, dogs may not show antibody responses in the early and acute phases of infection, and serum antibodies are lacking. Additionally, vaccination-induced antibodies have been described in some cases to last until 12 months after vaccination [24]. Further, under field conditions, it might even be possible that the encounter with *L*. spp. organisms in vaccinated individuals leads to an increase in vaccination-induced antibodies prolonging the seropositivity. The European Consensus statement from 2015 thus recommends the use of paired MAT titers with 4 weeks in between considering the vaccination history when possible [16]. PCR tests are mostly based on the *lipL32*-gene specific for pathogenic *L*. spp. [25, 26] or a 16S rRNA gene fragment [27], whereby both blood and urine should be tested from the same patient. Blood is only positive during leptospiremia until around 10 days and the urine becomes positive earliest after approximately 8 days postinfection [16, 28]. None of the PCR test methods allow the identification of the infecting leptospiral serogroup or serovar. Instead, identification of serovars is possible *via* molecular typing—depending on the leptospiral genomospecies—using either the multi locus variable number of tandem repeat analysis (MLVA/VNTR [29]), multispacer sequence typing (MST; [30, 31, 32]), or the multi-locus sequence typing (MLST; [33, 34]). However, all these tests display limited suitability in clinical settings as they require large amounts of leptospiral DNA and may lack precise distinction of reproducibility [29, 32]. Therefore, at the moment a combination of MAT and PCR of both blood and urine are the fastest and most reliable methods for routine diagnostics of leptospirosis in dogs as the information pertaining to the disease-causing serovar does not impact the treatment regime [35]. Dogs diagnosed with leptospirosis should be treated with two different antimicrobial agents aiming for the extra- and intra-cellular leptospiral organisms and depending on the affected organ supportive care might be necessary [16, 35]. As diagnosis is difficult and clinical signs can be severe with high chance of a fatal outcome of disease, the protection of dogs with efficient and epidemiologically relevant vaccines is strongly recommended. Accordingly, the vaccination of dogs against leptospirosis is considered a core vaccination in many European countries [36, 37]. Vaccination schedules recommend the primary vaccination protocol with two injections 4 weeks apart starting at 7 weeks of age and yearly revaccination [38]. Vaccines for veterinary use are multivalent, whole-cell bacterins inducing mostly a humoral immune response against lipopolysaccharides on the surface of the spirochete with a duration of immunity of around 1 year [39]. Furthermore, the immunity is restricted to serovars with related agglutination antigens, thus only limited cross-immunity between different serogroups exists [39, 40]. This lack of cross-reactivity between serogroups is a problem when considering that around 17 pathogenic leptospiral species with over 300 different serovars have been described [41, 42]. Due to this restriction, precise insight into the epidemiological occurrences at the vaccinee's location is needed. There are various vaccines against canine leptospirosis on the European market which protect against either two serogroups (*L*. Canicola and *L*. Icterohaemorrhagiae), three serogroups (*L*. Canicola, *L*. Icterohaemorrhagiae, and *L*. Grippotyphosa), or four serogroups (*L*. Canicola, *L*. Icterohaemorrhagiae, *L*. Grippotyphosa, and *L*. Australis) [38]. The latest seroprevalence study using MAT to screen leptospiral serogroups appearing in dogs from Europe has, to the best of the authors' knowledge, been reported before 2017 [43, 44, 45, 46, 47, 48, 49, 50]. It is, however, difficult to compare that these reports as various serum antibody titers were used as a threshold, dogs with or without vaccination were excluded, and different serovars were tested. In contrast, the present study's aim is a standardized screening of a collection of purified DNA extracted from canine field samples (blood and urine, *n* = 239) collected from dogs suspected of leptospirosis in Europe and found positive by applying a *lipL32* PCR. For this matter, a molecular-serogroup-typing PCR (MST PCR) was developed to differentiate *L*. serogroups by first identifying the leptospiral genomospecies using 16S rRNA gene PCR and Sanger sequencing, and thereafter typing the serogroup using a newly developed MST PCR based on specific primers amplifying the targeted *rfb* gene locus coding the LPS biosynthesis.

## 2. Materials and Methods

### 2.1. Development of the MST PCR

The first aim of this work was the development of the MST PCR for typing *L*. spp. Therefore, eight *L*. serogroups considered the most prevalent serogroups in Europe and the US according to recent reports [43, 44, 45, 46, 47, 48, 49, 50] were targeted (*L*. Australis (AUS), *L*. Autumnalis (AUT), *L*. Canicola (CAN), *L*. Grippotyphosa (GRI), *L*. Icterohaemorrhagiae (ICT), *L*. Pomona (POM), *L*. Pyrogenes (PYR), and *L*. Sejroe (SEJ)). These serogroups are found in pathogenic *L. interrogans* (all eight serogroups), *L. kirschneri* (AUT, GRI, ICT, POM, and PYR), and *L. borgpetersenii* (PYR and SEJ) species [1]. The aim of the PCR was a specific amplification of each serogroup, requiring the design of primers in regions specific to each serogroup. The serovar and serogroup are defined by the leptospiral lipopolysaccharide (LPS); thus, the search for locations to which primers can adapt was focused on the *rfb* gene locus responsible for LPS biosynthesis. Therefore, one reference strain representative for each of the 11 serogroups described above was selected and the according fasta file was downloaded from the NCBI Genomes database [51]. The 11 strains were selected based on the level of sequence assembly: complete chromosomes when available, otherwise, strains with a smaller number of scaffolds (Table 1).

### 2.2. Blasting *rfb* Gene Locus against *L. interrogans*

Identification of the *rfb* gene loci of the different serogroups was performed by blasting the well-characterized *rfb* gene locus of *L. interrogans* serovar Copenhageni str. Fiocruz L1130 against the 10 other strains using NCBI BLASTN [52]. The complete sequences of *rfb* gene loci were identified for almost all serogroups except for CAN and POM serogroups. For the POM serogroup, the *rfb* gene locus was divided into three different scaffolds which were assembled to reconstruct its complete *rfb* gene locus. For the CAN serogroup, the sequence of the *rfb* gene locus was complete but not annotated, thus, a coding sequence prediction was performed for the comparative study (Table 1).

### 2.3. Design of Primer Pairs in the Hypervariable Region of the *rfb* Gene

**Figure 1:**
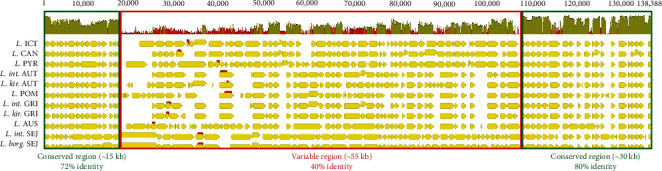
Alignment of *rfb* gene loci amongst the eight serogroups from three species. The highly conserved regions are highlighted by green frames, while the variable region is highlighted by a red frame. AUS, *Leptospira* (*L*.) Australis; AUT, *L*. Autumnalis; CAN, *L*. Canicola; GRI, *L*. Grippotyphosa; ICT, *L*. Icterohaemorrhagiae; POM, *L*. Pomona; PYR, *L*. Pyrogenes; SEJ, *L*. Sejroe; *int*., *L. interrogans*; *kir*., *L. kirschneri*; *borg*., *L. borgpetersenii*.

The *rfb* gene loci of the 11 strains were aligned using the Geneious software (Biomatters Inc., Boston, USA), allowing the identification of three regions: the first 15 kb are conserved between the strains of the eight serogroups at 72% of similarity, followed by 55 kb of variable regions sharing 40% of similarity, and 30 kb conserved at 80% of similarity at the end of the locus (Figure 1).

Within the variable region (55 kb), the first 12 kb are hypervariable with a similarity of 31% between the strains. This hypervariable region was then selected to design primers specific to amplify each leptospiral serogroup *via* PCR (Table 2; amplicons are highlighted in red in Figure 1). The primers were identified using the SnapGene software, version 5.0.1 (GSL Biotech LLC, San Diego, USA).

*In silico* analysis of the sequence of O-antigen clusters in 11 representative strains (Fiocruz L1-130, PigK151, LP101, 1051, LJ178, 56609, RM52, LC82-25, UP-MMC-NIID LP, Norma, and L550) displayed a possible serogroup-specificity of several glycosyltransferase and sugar synthesis genes (Table S1). Consequently, primers for the serogroup-specific PCR were designed based on these genes (Table 2). Newly designed primers were tested bioinformatically to ensure serogroup specificity. Therefore, all primers were tested using NCBI BLASTN [52].

### 2.4. Experimental Validation of Primers Using Reference Strains

Primers developed and validated bioinformatically were produced by Eurofins Genomics GmbH (Ebersberg, Germany). These primers were then experimentally validated using 27 reference strains belonging to the serogroups of interest from the VetAgro Sup collection (Marcy l'Etoile, France, Table 3). To assess the appropriate annealing temperature for the individual serogroups' primers, PCRs were tested at the temperatures 54, 56, 58, and 60°C. Finally, the temperature was chosen, which displayed the best sensitivity for the respective serogroup (Table 2). Endpoint PCR amplification was performed in 50 *μ*l volumes containing 5 *µ*l of 10 × PCR buffer, 1 mM MgCl_2_, 200 mM deoxynucleotide triphosphates, 0.5 *μ*M of each primer, 1 U Hot Star DNA polymerase (QIAGEN GmbH, Hilden, Germany), 5 *µ*l DNA sample, and PCR-grade water.

Thermal PCR conditions were as follows: initial denaturation at 95°C for 15 min, followed by 35 cycles of 30 s at 94°C denaturation, 30 s annealing at temperatures varying according to the annealing temperatures of the primer pairs (listed in Table 2), 1 min elongation at 72°C, and 10 min as a final elongation at 72°C.

Amplification products were analyzed by electrophoresis in 1.5% (wt/vol) agarose gels at 100 V for 60 min in 1 × TAE. By analyzing the PCR products, we excluded primer pairs that generated false-positive results for strains belonging to other serogroups and selected primer pairs that were able to discriminate as many strains belonging to the same serogroups as possible.

### 2.5. Establishment of the Final PCR Protocol and use of the Newly Developed MST PCR Protocols on Field Isolates

Biological samples from 239 field dogs originating from 158 clinics across 12 European countries were submitted to IDEXX Laboratories (Kronwestheim, Germany) for leptospirosis diagnosis based on clinical suspicion between 2017 and 2020. Total DNA was extracted from 70 blood and 169 urine samples. For this retrospective study, only available DNA residuals of samples sent to IDEXX Laboratories were used, no animals were kept or sampled for this experiment.

Diagnosis confirmation was performed at IDEXX Laboratories (Kornwestheim, Germany) using a *lipL32* qPCR, specific for pathogenic *L*. spp. Briefly, total DNA was extracted from blood samples by using the QIAamp® DNA Blood BioRobot® MDx kit (QIAGEN GmbH) and from urine samples by applying the QIAamp Viral RNA Mini Kit (QIAGEN GmbH) according to manufacturer's instructions [32]. Detection of *L*. spp. DNA was achieved by qPCR using the LightCycler 480 (Roche Deutschland Holding GmbH, Mannheim, Germany) with proprietary forward, reverse primers, and hydrolysis probes targeting *lipL32*-gene (accession number AF245281.1). The PCR assay was designed to have a reproducible average analytical sensitivity of 10 DNA molecules per reaction. Samples were regarded as positive when the Ct-value was below 40.

Samples displaying a positive signal in the *lipL32*-gene PCR (*n* = 239) were sent to the Research & Development Centre of Boehringer Ingelheim in Lyon (France), where the subsequent methods were applied.

*L*. genomospecies was identified using a 16S rRNA Sanger sequencing. Therefore, 3 *µ*l of the previously isolated DNA were amplified using the primers lepto-A 5′-GGCGGCGCGTCTTAAACATG-3′ and lepto-B 5′TTCCCCCCATTGAGCAAGATT-3′ described by Mérien et al. [27], with 40 PCR cycles at 58°C. Only amplified samples were further analyzed, while samples displaying a negative signal were excluded. The PCR products of positive samples (*n* = 226) were sent for Sanger sequencing to Eurofins Genomics GmbH (Ebersberg, Germany). Additionally, reference strains for *L. interrogans*, *L. kirschneri*, and *L. borgpetersenii* were sequenced. Sequences obtained from Eurofins Genomics GmbH were checked for quality using FastQC [53]. 16S rRNA sequences were aligned to an internal database of sequenced strains and NCBI genomes. Alignments from the field samples were then compared to the sequences of the reference strains using SnapGene software, version 5.0.1 (GSL Biotech LLC), and NCBI BLAST software [52] for species identification. Depending on the identified *L*. genomospecies (i.e., *L. interrogans*, *L. kirschneri*, and *L*. *borgpetersenii*), the protocol for the MST PCR was adapted to search for the most probable serogroups to spare volumes of clinical samples, as indicated in Table 4. PCR conditions were described above.

### 2.6. Data Analysis

Data for MST PCR were collected and examined using Microsoft Excel (Microsoft 365, Microsoft Corporation, Redmond, USA). Figures were created using Geneious software (Biomatters Inc.), OriginPro 2021b (Origin Lab, Northampton, Massachusetts, USA), and Adobe Illustrator S6 version 16.0.0 (Adobe Inc., San José, CA, USA). The map was created using mapchart (https://www.mapchart.net/index.html, accessed 05/30/2023).

## 3. Results and Discussion

### 3.1. Development of the New PCR Protocol

The primers designed for the MST PCR were validated in the first step by identifying suitable primer pairs with bioinformatical tools (i.e., NCBI BLASTN [52]). In the second step, established PCR protocols and primers were validated with *L*. spp. reference strains available at VetAgro Sup (France).

For bioinformatic validation, the efficiency of the designed primers to other strains of the same serogroups was tested by first downloading all genomes belonging to the studied serogroup, available on the NCBI database. Designed primers were then blasted against these 226 genomes using NCBI BLASTN [52]. BLAST results perfectly matched the 11 primer pairs and the expected serogroup for 217 out of 226 genomes (Table 5). Among the 217 genomes, *L. kirschneri* and *L. borgpetersenii* POM serogroup genomes matched with POM primers designed on *L. interrogans* and *L. santarosai* genomospecies. The *L*. Sejroe serogroup matched with SEJ primers designed on *L. interrogans* and *L. borgpetersenii* (Table 2), extending the application of the designed primers. The unexpected mismatches with 14 genomes were investigated. Four NCBI genomes display an incomplete *rfb* gene locus due to flawed sequencing assemblies, explaining the absence of primer match. Five NCBI genomes matched with primer pairs of unrelated serogroups. The obtained typing results were consistent with the matches found with the primer pairs designed in this study, suggesting an annotation error on the database, and confirming the specificity of the developed tool.

**Figure 2:**
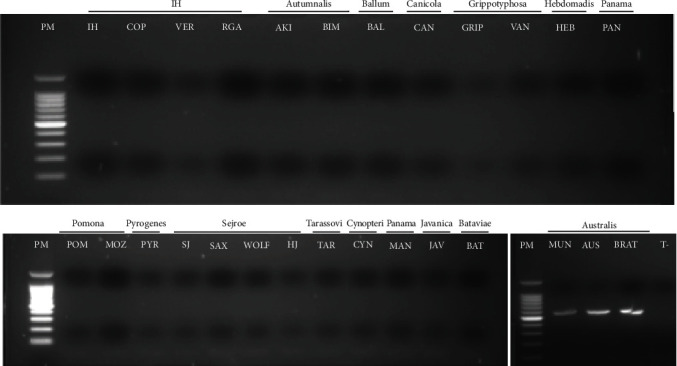
Agarose gel images of PCR amplification with AUS primers on 26 DNA samples extracted from strains of various serogroups to test efficiency and specificity. The row above the gel image depicts the serovar tested, while the bracket above indicates the serogroup of *L*. spp. PM, 100-pb ladder invitrogen 15628019; IH, *L. interrogans* Icterohaemorrhagiae Icterohaemorrhagiae 16; COP, *L. interrogans* Icterohaemorrhagiae Copenhageni Winjberg; VER, *L. interrogans* Icterohaemorrhagiae Icterohaemorrhagiae Verdun; RGA, *L. interrogans* Icterohaemorrhagiae Icterohaemorrhagiae RGA; AKI, *L. interrogans* Autumnalis Autumnalis Akiyami; BIM, *L. kirschneri* Autumnalis Bim 1051; BAL, *L. borgpetersenii* Ballum Castellonis Castellon 3; CAN, *L. interrogans* Canicola Canicola Hond Utrecht IV; GRIP, *L*. *kirschneri* Grippotyphosa Grippotyphosa Moskva V; VAN, *L. kirschneri* Grippotyphosa Vanderhoedoni Kipod 179; HEB, *L. interrogans* Hebdomadis Kremastos Kremastos; PAN, *L. noguchii* Panama Panama CZ 214 K; POM, *L. interrogans* Pomona Pomona Pomona; MOZ, *L. kirschneri* Pomona Mozdok 5621; PYR, *L. interrogans* Pyrogenes Pyrogenes Salinem; SJ, *L. borgpetersenii* Sejroe Sejroe M 84; SAX, *L. interrogans* Sejroe Saxkoebing Mus 24; WOLF, *L. interrogans* Sejroe Wolffi 3705; HJ, *L. interrogans* Sejroe Hardjo Hardjoprajitno; TAR, *L. borgpetersenii* Tarassovi Tarassovi Perepelitsin; CYN, *L. kirschneri* Cynopteri Cynopteri 3,522 C; MAN, *L. noguchii* Panama Mahnus TRVL/CAREC137774; JAV, *L. borgpetersenii* Javanica Javanica Veldrat Baravia 46; BAT, *L. interrogans* Bataviae Bataviae Van Tienen; MUN, *L. interrogans* Australis Muenchen Muenchen C90; AUS, *L. interrogans* Australis Australis Ballico; BRAT, *L. interrogans* Australis Bratislava Jez-Bratislava; T-, nuclease-free water.

The experimental validation step using 28 reference strains belonging to the serogroup and species of interest was performed thereafter. Primers amplified all DNA serogroup specific as shown on the agarose gel image when amplified with the primers AUS (Figure 2), AUT_int (Figure S1), AUT_kir (Figure S2), CAN (Figure S3), GRI (Figure S4), ICT (Figure S5), POM-2 (Figure S6), PYR-2 (Figure S7), and SEJ (Figure S8).

### 3.2. Serogroup Detection Study in Europe Using the Newly Developed MST PCR on Field Isolates

Biological samples from 239 field dogs (blood: *n* = 70; urine: *n* = 169) positive for leptospirosis originating from 158 clinics across 12 European countries between 2017 and 2020 were selected for the MST PCR. The highest number of samples was available from Germany (*n* = 89), followed by Italy (*n* = 40), the United Kingdom (*n* = 38), Spain (*n* = 23), the Netherlands (*n* = 17), France (*n* = 16), Denmark (*n* = 5), Austria (*n* = 4), the Czech Republic (*n* = 3), Poland (*n* = 2), and Norway (*n* = 1). Information on the sampled individuals was not available in this study as only residual DNA was anonymously obtained. However, in future studies, more information on the sampled individuals such as breed, environment, clinical signs, and contact with infectious sources or hosts should urgently be addressed.

**Figure 3:**
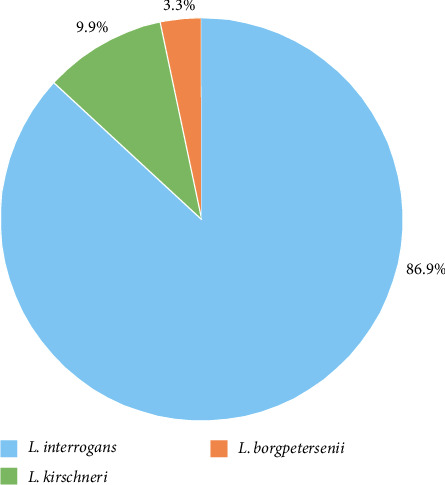
Fractions of *Leptospira* (*L*.) genomospecies *L. interrogans*, *L. kirschneri*, and *L. borgpetersenii* detected in European regions originating from 213 DNA samples containing leptospiral DNA according to the similarity of the 16S rRNA gene.

The 16S rRNA gene PCR and the sequencing of the 16S rRNA gene produced a total of 213 samples (92.5%) containing DNA from *L*. spp., while for 18 samples either the PCR amplification or the sequencing of the 16S rRNA gene was not successful. Of these, 213 samples with a positive result, identified species were in large majority *L. interrogans* (87.0%; *n* = 185), followed by *L. kirschneri* (9.9%; *n* = 21), and *L. borgpetersenii* (3.3%; *n* = 7; Figure 3). Eight samples showed DNA signals that were not related to a *L*. spp. The eight samples having DNA that was not associated with leptospiral species showed the limitation of the *lipL32* and 16S rRNA PCR. However, the false-positive rate of 3.4% is comparable to that found in the literature [54]. In these *L*. spp. negative samples, the species identified most frequently by BLASTing the 16S rRNA gene sequences was *Collinsiella intestinalis*. The amplification of further nonleptospiral bacteria with a 16S PCR has already been described by other authors [55]. This occurs most frequently when amplifying urine samples, as a higher proportion of other “contaminating” bacteria can occur in these samples. Accordingly, when a higher proportion of “contaminating” bacteria is sequenced, this fraction will consequently constitute the highest number of reads after sequencing. Thus, these samples might not be false-negative but rather the leptospiral fraction can be underrepresented after Sanger sequencing and the identification is not possible. Indeed, of the five samples containing *Collinsiella intestinalis* four originated from urine samples and might thus display a contamination. Still, the reason for the high number of *Collinsiella intestinalis* detected in this study is not clear to the authors.

**Figure 4:**
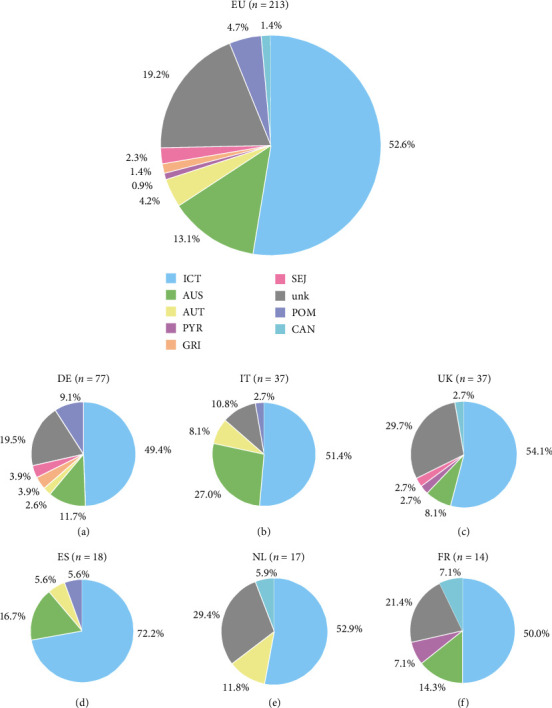
Serogroups (in %) detected in all European regions (EU) and individual countries using MST PCR from: (a) DE, Germany; (b) IT, Italy; (c) UK, United Kingdom; (d) ES, Spain; (e) NL, Netherlands; (f) FR, France. ICT, *Leptospira* (*L*.) Icterohaemorrhagiae; AUS, *L*. Australis; AUT, *L*. Autumnalis; PYR, *L*. Pyrogenes; GRI, *L*. Grippotyphosa; SEJ, *L*. Sejroe; unk, unknown; POM, *L*. Pomona; CAN, *L*. Canicola.

According to the protocol described above, for samples positive for leptospiral DNA (*n* = 213), a molecular typing protocol was used to identify the serogroup using various PCR protocols and primers (Tables 2 and 4). This molecular typing was successful for 172 samples (80.8%) and both species and serogroup were annotated for these samples. No serogroup was allocated to 41 samples (19.2%; Figure 4). Of these unknown serogroups, 34 samples belonged to *L. interrogans*, five to *L. kirschneri*, and two to *L. borgpetersenii*.

As only the most prevalent serogroups in Europe, based on the available literature, were tested with the MST PCR protocol, it might be possible that the remaining leptospiral DNA originated from a serogroup not tested in this study. Certainly, this is a limitation of the developed protocol as the leptospiral genomospecies may contain a wide variety of serogroups. However, this is not only limited by the primer pairs applied for the MST PCR but further by the amount of leptospiral DNA available in the sample. Nevertheless, current molecular typing methods that are used (i.e., MST, MLST, or MLVA) are prone to similar limitations [56] and identify only a small array of certain serovars [34, 57, 58, 59]. Especially the MLST and MLVA depend on a large volume of extracted DNA with sufficient DNA concentrations that in many cases are not available. Most studies used for leptospiral epidemiology in veterinary medicine are based on microagglutination testing (MAT) applied to serum antibodies. This test allows the identification of serogroups but hardly differentiates between vaccinated and infected dogs. However, the main limitation of MAT is its lack of standardization and compatibility between different laboratories [23]. Additionally, the compatibility between studies is problematic as different cut-off titers for the MAT are used [60]. Consequently, the MST PCR does not solve the problem of a limited number of serogroups tested but provides a protocol pertaining to standardization, differentiation of vaccinated and infected animals, and to some extent the limited availability of leptospiral DNA. In recent years, methodologies using whole genome sequencing (WGS) and especially core genome multilocus sequence typing (cgMLST) have advanced and allow even more precise identification of a broader spectrum of serogroups and additionally serovars of pathogenic, intermediate, and saprophytic leptospiral species through bioinformatical methods [56, 61]. Nevertheless, and to the best of our knowledge, cgMLST applications on samples with animal origins are not available yet, and before WGS can be applied, organisms need to be cultivated in media. As the cultivation of leptospiral organisms is laborious, the MST PCR introduced in this work might be suitable for future epidemiological projects, providing better standardization and interlaboratory comparability than the methods described above. Further developments of WGS methods, i.e., the hybridization capture method described by Grillova et al. [62] will probably allow a better understanding of epidemiological situations in the far future. Lastly, a thorough comparison of all these methods is needed to gain a better understanding of the benefits and limitations of these methods. This is as described above hardly possible and consequently, the authors were not able to conduct this comparison.

Focusing on the 172 samples obtained from dogs throughout Europe that were successfully typed to a leptospiral species and serogroup, the highest prevalence by MST PCR was observed for serogroup ICT, followed by AUS, POM, and AUT in descending order (Figure 4).

For Germany, Italy, the United Kingdom, Spain, the Netherlands, and France, there were more than 10 samples available for the MST PCR per county. Therefore, the prevalence of *L*. spp. for these countries is described in detail in Figure 4. For Denmark (*n* = 4), Austria (*n* = 4), Czech Republic (*n* = 2), Poland (*n* = 1), Norway (*n* = 1), and Slovakia (*n* = 1), sample numbers were too low to show a realistic prevalence for these countries individually.

It needs to be emphasized that most comparisons to the literature below were done with studies based on antibody levels against leptospiral serogroups and serovars detected with the MAT. Additionally, in some of these studies, vaccinated animals were included or excluded as were serogroups found in the vaccination. Thus, we compare the seroprevalence of antibodies against *L*. spp. in dogs with the prevalence of DNA extracted from leptospiral organisms in blood and urine samples. In some cases, a comparison was performed with serovars and according to serogroups from MST, MLST, or MVLA. Furthermore, none of these studies tested all serogroups, serovars, or even species due to the methodological limitations described above [56]. Moreover, it is important to note, that this study does not focus on the general epidemiological prevalence of leptospirosis in European dogs, but rather on the prevalence in diseased dogs. Consequently, by writing prevalence in the paragraphs below, we do refer to the prevalence in populations of diseased dogs.

**Figure 5:**
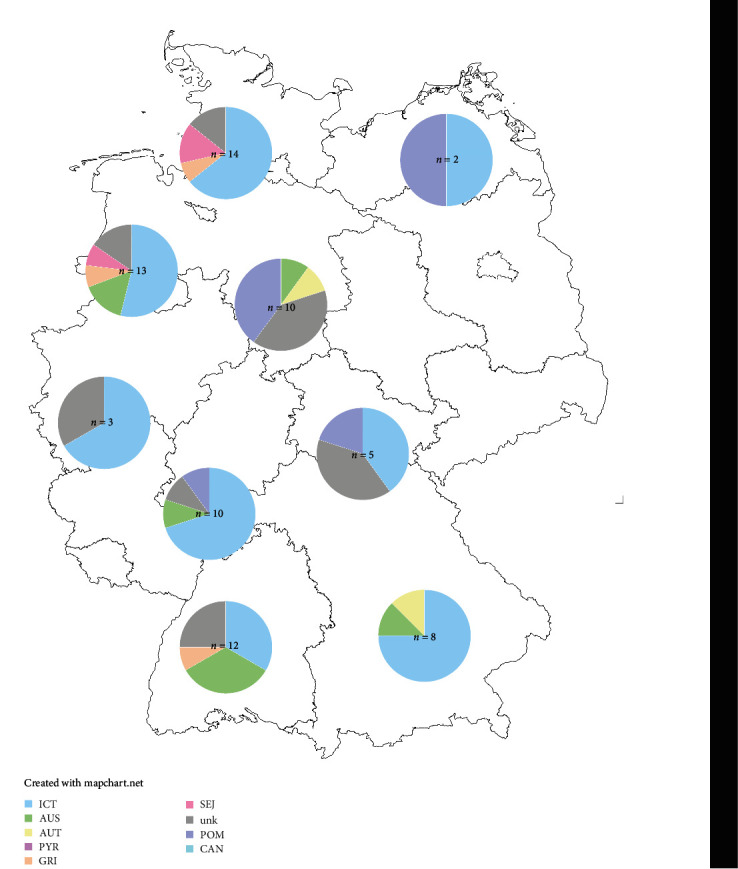
Prevalence of leptospiral serogroups in German regions detected by MST PCR associated with zip codes. ICT, *Leptospira* (*L*.) Icterohaemorrhagiae; AUS, *L*. Australis; AUT, *L*. Autumnalis; PYR, *L*. Pyrogenes; GRI, *L*. Grippotyphosa; SEJ, *L*. Sejroe; unk, unknown; POM, *L*. Pomona; CAN, *L*. Canicola.

Germany was the country from which most samples containing leptospiral DNA (*n* = 77) were available. The samples were received from all over Germany allowing a detailed description of the epidemiology of all regions (Figure 5). Here, the most prevalent serogroups were ICT, followed by AUS, POM, GRI, and SEJ in descending order (Figure 4(a)). To our knowledge, the latest data available for German dogs have been published by Llewellyn et al. [46] using the *lipL32* PCR, MLST, and MAT, and testing samples originating from dogs living in the Upper Bavaria region. Seroprevalence data produced with a MAT using an antibody titer cut-off equal to or higher than 1 : 100 revealed the following descending order: ICT, GRI, AUS, CAN, SEJ, AUT, and POM. While here in this study, ICT is the serogroup with the highest prevalence, the prevalence for GRI and CAN are considerably lower (Figure 4(a)). On the contrary, the serogroup prevalence for AUS and for POM was slightly or strongly higher than the prevalence observed by Llewellyn et al. [46], respectively, while for SEJ, it was nearly the same. A clinic in Berlin described in 2013 the following ranking in seroprevalence in northern parts of Germany: AUS, GRI, and POM [44]. A high prevalence for serogroup POM is still observed in the northeastern and central regions of Germany, while GRI is only rarely observed in the northwest of Germany (Figure 5). The low detection rate of serogroups GRI and CAN might originate from the efficacy of leptospirosis vaccination with vaccination coverage of around 50% in German dogs [63]. However, as GRI is a serogroup that occurs mostly when the responsible reservoir host (i.e., different species of voles, mice, and shrew, as well as hedgehogs [31, 64, 65]) is present and has been associated with a more rural environment [66], the decrease could simply originate from a higher urbanization rate. In general, the background on why some serogroups still circulated in the same number of dogs or even increased while others decreased remains unclear. The vaccination of dogs against leptospirosis is considered a core vaccine in Germany [37]. A high vaccination coverage in general is important when considering the One-Health concern.

The second highest number of serum samples containing leptospiral DNA was available from Italy (*n* = 37). Here, as in Germany, most samples contained the serogroup ICT, followed by AUS and AUT (Figure 4(b)). Previous reports from Italy agree with ICT being the most seroprevalent serogroup [67]. However, Grippi et al. [67] detected CAN as the second most seroprevalent serogroup when testing with the MAT, followed by AUS. Further reports describe AUS as the serogroup with the highest seroprevalence, followed by ICT and either CAN [47] or POM [68]. Not described in previous reports was the high prevalence of AUT (Figure 4(b)), while CAN was not detected in the work presented here. Another study conducted with both MAT and MLST/MLVA found ICT as the most prevalent serogroup followed by AUS, POM, and SEJ [69]. Similarly, as described here, the serogroup CAN has not been detected in Italy [69]. Since the other groups did not test for DNA or antibodies against AUT, this probably explains the discrepancies in the results presented here. To our knowledge, information regarding the assignment of the leptospirosis vaccine as core vaccination and on the vaccination quotes was not available.

Over half of the samples from the United Kingdom (*n* = 37) were assigned to the serogroup ICT, as described for Germany and Italy (Figure 4(c)). This serogroup was followed by AUS. Thereafter, the serogroups PYR, SEJ, and CAN follow at similar prevalence (Figure 4(c)). Previous publications from the United Kingdom describe antibodies against the serogroup ICT to be the most prevalent, followed either by CAN, AUS, and SEJ [19], or SEJ and AUS [70]. In the UK, vaccination against leptospirosis is considered a core vaccine with around half of the dog population being vaccinated yearly [71]. Comparable to mainland Europe, the vaccines contain the serogroups ICT, GRI, CAN, and the L4 polyvalent vaccine AUS in addition. Interestingly, compared to Germany and Italy, even though the same vaccines are used, and the vaccination quote is comparable to Germany, there are still some infections with the CAN serogroup observed in the United Kingdom. The numbers are similar but slightly lower, compared to a publication from the 1990s where 3.6% of dogs displayed culturable leptospiral organisms [72]. This consistent prevalence of the serogroup CAN in the United Kingdom might originate from a mutation of the serogroup CAN described to have occurred between 1960 and 1970 [66].

From Spain, 18 samples were available of which the majority of DNA was assigned to ICT. The second-highest prevalence was observed for AUS, thereafter, AUT and POM followed (Figure 4(d)). To our knowledge, the most recent work on the seroprevalence of *L*. spp. in dogs was published in 2019. Using MAT, the highest seroprevalence was observed for serogroup ICT, followed by AUS, GRI, AUT, CAN, and SEJ [73]. While our results agree with ICT and AUS as the two most prevalent serogroups, we did not detect samples assignable to the serogroups GRI, CAN, and SEJ. Instead, we observed a prevalence of over 5% for serogroups AUT and POM. In Spain, the vaccination against leptospirosis is considered a noncore vaccine, and no publications pertaining to vaccination quotes are available.

Of the 17 samples available from the Netherlands, most samples were identified as ICT, followed by AUT and CAN (Figure 4(e)). To the best of our knowledge, there is no literature available on the seroprevalence of *L*. spp. or the vaccination quote in the Netherlands.

Lastly, 14 samples were available from France, with half of the samples assigned to serogroup ICT, followed by AUS, PYR, and CAN (Figure 4(f)). Previous studies described antibody reactions against serogroup AUS as the most prevalent, followed by PYR, GRI, and SEJ [45]. Interestingly, another study by Ayral et al. [45] tested the seroprevalence of ICT, however, did not detect this serogroup in the dog sera available. Another study with canine samples using the MAT looked only for antibody reactions against serovars not covered by vaccination until 2013. Representative serovars used for the MAT were the serogroups AUS, GRI, and SEJ [43]. In this study, antibody reactions against AUS were observed most frequently, followed by serogroups GRI and SEJ. A comparison of our results with this study is difficult, as many prevalent serogroups we identified were not tested by Renaud et al. [43].

Seroprevalences from other countries besides the ones tested have previously been published from Sweden (SEJ, ICT, AUT, and CAN [74]), Switzerland (AUS, ICT, CAN, GRI, POM, and AUT [75]), and Ireland (Ballum, AUS, POM, and SEJ [48]).

The main aim of the leptospirosis vaccination is the reduction of clinical signs and further protection from developing a persistent infection in the kidneys [60]. The latter aim ensures reduced renal shedding and thus prevents the transmission of leptospiral organisms to other animals and the human [76]. This protection might become especially important in European countries as an increase in leptospirosis cases due to climate change should be expected. As there is a lack of evidence for cross-reactivity between *L*. serovars, the vaccine should be expected to only protect against the serovars included in the respective vaccination. Thus, it is urgent to continuously monitor the epidemiology of canine *L*. spp. infections using a direct detection method. On the other hand, studies regarding the prevalence of *L*. spp. in other mammals provide helpful insight into the prevalence of the pathogen in maintenance hosts and allow insight into possible serogroups circulating in wildlife and possibly endangering dogs. Considering, the *L*. spp. prevalence displayed in this work, the most frequently observed serovars are ICT, AUS, POM, AUT, and SEJ. The vaccines currently used protect against the serovars ICT, CAN, GRI, and AUS. The serovars ICT, GRI, and AUS are maintained in rodents and small mammals throughout Europe that still display high seroprevalences [31, 47, 64]. The continuous survey of canine leptospirosis epidemiology throughout Europe is needed urgently. Thereby, the best option would be a Europe-wide joined approach to collect a sufficient number of samples from all European countries, considering dogs with and without suspected leptospirosis, applying the same diagnostic method and thus enabling the best survey of canine leptospirosis epidemiology.

## 4. Conclusions

Here, we present a novel MST PCR approach for the detection of leptospiral serogroups most prevalent in Europe. This MST PCR was validated with reference strains and field samples and can be used for clinical samples to conduct serogroup prevalence studies. In comparison to currently used methods, the MST PCR enables better standardization and requires less DNA volume. Additionally, as the MST PCR detects DNA of leptospiral organisms but not infection- or vaccination-induced antibodies, differentiation between vaccinated and infected dogs is possible. This MST PCR was used to assess the prevalence of *L*. serogroups in confirmed leptospirosis-positive dogs based on PCR with samples originating from several European countries. Most other studies focused on single countries using either the MAT or MLST/VNTR. In European countries with sufficient sample numbers available, the most prevalent serogroup was ICT followed by AUS. The prevalence of other serogroups (i.e., AUT, CAN, GRI, POM, PYR, and SEJ) differed strongly among different countries. However, when considering the European Union as a borderless trade zone, mostly lacking geographical borders such as rivers and mountains that are impassable by wildlife, a generalized European approach for leptospirosis vaccination of dogs might be most reasonable, especially when One-Health concerns are considered. In this regard, besides ICT and AUS, the serogroups POM and AUT seem to be emerging in some regions (Figure 4). The inclusion of a host-adapted serogroup (i.e., CAN) is recommended [66] and the prevalence in small mammals supports the further use of serogroups ICT, GRI, and AUS. Consequently, current L4 vaccines are relevant and should confer a high efficacy profile. Nevertheless, as the limited selection of serogroups available in the current vaccination will not protect against other serogroups not implemented in the L4 vaccines, finding ways to protect against a broader range of leptospiral serogroups remains an issue to be solved in the future.

## Figures and Tables

**Table 1:** Representative genome per serogroup and species of epidemiological relevance and *rfb* gene locus characteristics.

Sero-group	Species	Representative genome	Assembly level	Genbank accession	*rfb* locus	
					Size (kb)	Number of CDS
AUS	*L. interrogans*	Bratislava str. PigK151	Complete	CP011410	94.6	87
AUT	*L. interrogans*	Autumnalis str. LP101	127 scaffolds	AHNF02	90.7	79
	*L. kirschneri*	Bim str. 1051	36 scaffolds	AHML02	96.4	92
CAN	*L. interrogans*	Canicola str. LJ178	Complete	CP044509	100.8	88^*∗*^
GRI	*L. interrogans*	Linhai str. 56609	Complete	CP006723	90.6	78
	*L. kirschneri*	Grippotyphosa str. RM52	28 scaffolds	AHMJ02	93.3	81
ICT	*L. interrogans*	Copenhageni str. Fiocruz L1-130	Complete	AE016823	99.5	90
POM	*L. interrogans*	Pomona str. Kennewicki LC82-25	71 scaffolds	AHMK02	100.5^*∗∗*^	93^*∗∗*^
PYR	*L. interrogans*	Manilae str. UP-MMC-NIID LP	Complete	CP011931	102.3	93
SEJ	*L. interrogans*	Hardjo str. Norma	Complete	CP012603	84.4	83
	*L. borgpetersenii*	Hardjo-bovis str. L550	Complete	NC_008508	84.9	81

AUS, *Leptospira* (*L*.) Australis; AUT, *L*. Autumnalis; CAN, *L*. Canicola; GRI, *L*. Grippotyphosa; ICT, *L*. Icterohaemorrhagiae; POM, *L*. Pomona; PYR, *L*. Pyrogenes; SEJ, *L*. Sejroe.  ^*∗*^CDS (coding sequence) prediction was performed in this study using CLC genomics software (CLC Genomics Workbench version 12.0.3, QIAGEN GmbH, Hilden, Germany) because the GenBank file was not annotated.  ^*∗∗*^Approximative since *rfb* gene locus is an assembly of three scaffolds.

**Table 2:** Primers developed for the identification of leptospiral serogroups by MST targeting the eight most prevalent serogroups in Europe according to literature.

Sero-group	Species	Name	Sequence 5′–3′	Annealing temperature (°C)	Amplicon size (bp)
AUS	*L. interrogans*	Aus_Fw	AGGGTTCATTGGATTTCATCTAAC	58	557
		Aus_Rv	GATGAGGAGCCATATCAGGTC		

AUT	*L. interrogans*	Aut_int_Fw	AGTTTGCACTTAGATTGAATCC	54	767
		Aut_int_Rv	CCCTCCCTCTCTTGTAATTTCTTGC		
	*L. kirschneri*	Aut_kir_Fw	ATAATAGATCATCACAGCTCACAGGTCG	60	209
		Aut_kir_Rv	GCCACGCTCAACGATAGATTGAATTGC		

CAN	*L. interrogans*	Can_Fw	AAGTTATAGAGTTCGGACCGG	58	488
		Can_Rv	CTGCAACGGTTGAATGATACTATC		

GRI	*L. interrogans* ^*∗*^	Gri_int_Fw	AGGAGATGTGACCTACATTTTGTC	58	522
		Gri_int_Rv	TTCACAATGTCCATTGCTTCTCC		
	*L. kirschneri*	Gri_kir_Fw	GACGTGGGGACAAAACATATGTC	60	533
		Gri_kir_Rv	TACAGCAGCAGCAACATCCGTAG		

ICT	***L. interrogans*** *L. kirschneri*	Ict_Fw	TGGCCTTGAAATTGGGCCATAAC	60	513
		Ict_Rv	ACCTAAACCAGCCTCTTCAAACC		

POM-2^+^	***L. interrogans*** *L. kirschneri*	Pom-2_Fw	AATCTCAGGAAGTATCTTCAATAC	56	718
		Pom-2_Rv	CATGCGCTGTATTATCAATAATTG		

PYR-2^+^	*L. interrogans*	Pyr-2_Fw	TGGCTTCTATTGCAAATCAGAATGG	56	580
		Pyr-2_Rv	CTCACCGGATATGCTCCTTTATGC		

SEJ	***L. interrogans*** ***L. borgpetersenii***	Sej_Fw	TGCATGCTTTGCTTCGATTAATTAAG	56	328
		Sej_Rv	GTTCGATACATTTCATTTAAGCTAG		

AUS, *Leptospira* (*L*.) Australis; AUT, *L*. Autumnalis; CAN, *L*. Canicola; GRI, *L*. Grippotyphosa; ICT, *L*. Icterohaemorrhagiae; POM, *L*. Pomona; PYR, *L*. Pyrogenes; SEJ, Sejroe; Fw, forward; Rv, reverse.  ^*∗*^*L. interrogans* GRI primers were tested in the validation study; however, due to the rare observation of this species serogroup in dog samples, it was not used in the protocol for field samples due to restricted DNA amounts. Bold letters indicate, on which *L*. spp. the primer originally was established. If more than one species is indicated the primer worked with both species in the PCR protocol and the case of SEJ the DNA sequence for *L. interrogans* and *L. borgpetersenii* was the same. ^+^For primer POM-2 and PYR-2, two previous primer pairs were designed (POM-1: Fw: 5′ACACTTTATGGGAAGAACACG-3′ and Rv: 5′TCAGGCAACAATCTACTTGGTG3′; PYR1: Fw: 5′ TTGGTGGACGTAGACTCTGAG3′ and Rv: 5′ACTCTTCCCTTCTATCAACTTG3′) that produced unspecific amplification products.

**Table 3:** Reference strains used for experimental validation of primers.

Serogroup	Species	Serovar	Strain
Australis	*L. interrogans*	Muenchen	Muenchen C90
Australis	*L. interrogans*	Australis	Ballico
Australis	*L. interrogans*	Bratislava	Jez-Bratislava
Autumnalis	*L. interrogans*	Autumnalis	Akiyami
Autumnalis	*L. kirschneri*	Bim	1051
Ballum	*L. borgpetersenii*	Castellonis	Castellon 3
Bataviae	*L. interrogans*	Bataviae	Van tienen
Canicola	*L. interrogans*	Canicola	Hond utrecht IV
Canicola	*L. kirschneri*	Galtoni	LT1014
Cynopteri	*L. kirschneri*	Cynopteri	3522 C
Grippotyphosa	*L. kirschneri*	Grippotyphosa	Moskva V
Grippotyphosa	*L. kirschneri*	Vanderhoedoni	Kipod 179
Hebdomadis	*L. interrogans*	Kremastos	Kremastos
Icterohaemorrhagiae	*L. interrogans*	Icterohaemorrhagiae	19
Icterohaemorrhagiae	*L. interrogans*	Icterohaemorrhagiae	RGA
Icterohaemorrhagiae	*L. interrogans*	Icterohaemorrhagiae	Verdun
Icterohaemorrhagiae	*L. interrogans*	Copenhageni	Winjberg
Javanica	*L. borgpetersenii*	Javanica	Veldrat batavia 46
Panama	*L. noguchii*	Panama	CZ 214 K
Panama	*L. noguchii*	Magnus	TRVL/CAREC137774
Pomona	*L. interrogans*	Pomona	Pomona
Pomona	*L. kirschneri*	Mozdok	5621
Pyrogenes	*L. interrogans*	Pyrogenes	Salinem
Sejroe	*L. borgpetersenii*	Sejroe	M 84
Sejroe	*L. interrogans*	Saxkoebing	Mus 24
Sejroe	*L. interrogans*	Wolffi	3705
Sejroe	*L. interrogans*	Hardjo	Hardjoprajitno
Tarassovi	*L. borgpetersenii*	Tarassovi	Perepelitsin

**Table 4:** MST PCR according to the *L*. genomospecies identified by Sanger sequencing.

*L*. genomospecies identified by sanger sequencing	First PCR	Second PCR	Third PCR	Fourth PCR	Fifth PCR	Sixth PCR	Seventh PCR
*L. interrogans*	ICT	AUS	POM	CAN	AUT	SEJ	PYR
*L. kirschneri*	ICT	POM	GRI	AUT	PYR	—	—
*L. borgpetersenii*	SEJ	PYR	—	—	—	—	—

AUS, *Leptospira* (*L*.) Australis; AUT, *L*. Autumnalis; CAN, *L*. Canicola; GRI, *L*. Grippotyphosa; ICT, *L*. Icterohaemorrhagiae; POM, *L*. Pomona; PYR, *L*. Pyrogenes; SEJ, *L*. Sejroe.

**Table 5:** Validation of the MST PCR to identify the eight major serogroups on reference genomes and strains with either BLASTN against 217 NCBI genomes or PCR on 28 leptospiral reference strains' DNAs (Table 3) belonging to the targeted serogroups.

Primers	Number of characteristics tested with			Species	Bioinformatics (NCBI BLASTN)	Experimental (PCR)	Specificity
	NCBI genomes	DNA strains	Serovars				
AUS	8	3	5	*L. interrogans*	OK	OK	OK

AUT	7	2	4	*L. interrogans*, *L. kirschneri*	OK	OK	OK

CAN	8	3	3	*L. interrogans*, *L. kirschneri* ^*∗*^	OK	OK	OK

GRI	19	4	4	*L. interrogans* ^*∗*^, *L. kirschneri*	OK	OK	OK

ICT	113	4	4	*L. interrogans*, *L. kirschneri*	OK	OK	OK

POM-2	18	7	4	*L. interrogans*, *L. kirschneri*	OK	OK	OK

PYR-2	23	1	3	*L. interrogans*, *L. borgpetersenii*	OK	OK	OK

SEJ	21	4	10	*L. borgpetersenii*, *L. santarosai* ^*∗*^	OK	OK	OK

AUS, *Leptospira* (*L*.) Australis; AUT, *L*. Autumnalis; CAN, *L*. Canicola; GRI, *L*. Grippotyphosa; ICT, *L*. Icterohaemorrhagiae; POM, *L*. Pomona; PYR, *L*. Pyrogenes; SEJ, *L*. Sejroe.  ^*∗*^These species serogroups and primers were not tested further on the field samples in this project, but the primers would work if used.

## Data Availability

The sequences generated by Sanger sequencing that support the assignment of samples to a leptospiral genomospecies are available from the corresponding author, Reinhard K. Straubinger, upon reasonable request.

## Supplementary Materials

Supplementary Materials Figures S1–S8 and Table S1 are given in supplementary materials for comprehensive data and image analysis.
