# Supplementary material for: Detection and Identification of Pathogenic Leptospira spp. Serogroups in Europe between 2017 and 2020 Applying a Novel Gene-Based Molecular Approach
Source: Transbound Emerg Dis. 2024 Jun 11;2024:1101841. doi: 10.1155/2024/1101841 (PMC12020406; doi:10.1155/2024/1101841)
Supplement: Supplementary Materials — Figures S1–S8 and Table S1 are given in supplementary materials for comprehensive data and image analysis. [file 1101841.f1.docx]

**Detection and identification of pathogenic *Leptospira* spp. serogroups in Europe between 2017 and 2020 applying a novel gene-based molecular approach**

Supplementary Material

**Supplementary Table 1.** Genes targeted by the molecular serogroup typing method.

| Serogroup | Targeted gene | Product |
| --- | --- | --- |
| AUS | BRAT_RS11710 | Protein CapI |
| AUT | *L. interrogans*: LEP1GSC089_0828 | Glycosyltransferase |
|  | *L. kirschneri*: LEP1GSC046_3470 | Glycosyltransferase |
| CAN | ORF_21 | Methyltransferase type 12 |
| GRI | *L. interrogans*: LIL_12310 | Short chain dehydrogenase |
|  | *L. kirschneri*: LEP1GSC044_2689 | Short chain dehydrogenase |
| ICT | LIC12185 | Fucose synthetase |
| POM | LEP1GSC045_1476 | Glycosyltransferase |
| PYR | LIMLP_RS10815 | DegT/DnrJ/EryC1/StrS family aminotransferase |
| SEJ | *L. interrogans*: G436_1770 | D-alanine:D-alanine ligase |
|  | *L. borgpetersenii*: LBL_RS06650 | Class I SAM-dependent methyltransferase CDS |

AUS, *Leptospira* (*L.*) Australis; AUT, *L.* Autumnalis; CAN, *L.* Canicola; GRI, *L.* Grippotyphosa; ICT, *L.* Icterohaemorrhagiae; POM, *L.* Pomona; PYR, *L.* Pyrogenes; SEJ, *L.* Sejroe.


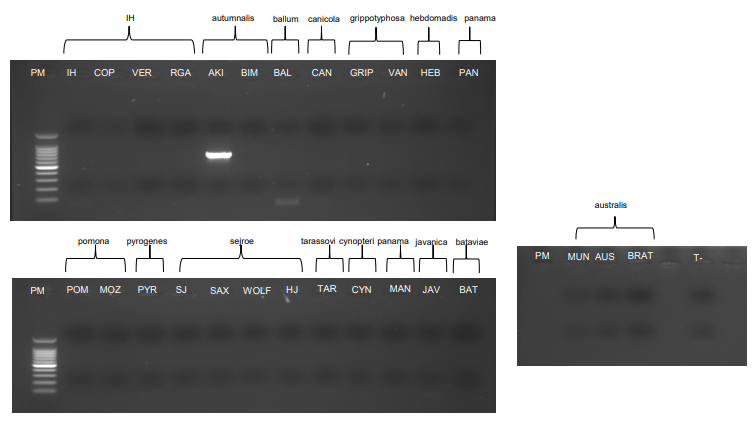


Supplementary Figure 1. Agarose gel images of PCR amplification with AUT_int primers on 26 DNA samples (indicated in Table 3) extracted from strains of various serogroups to test efficiency and specificity. The row above the gel image depicts the serovar tested, while the bracket above indicates the according serogroup of *L.* spp.

PM, 100-pb ladder Invitrogen 15628019; IH, *L.* *interrogans* Icterohaemorrhagiae Icterohaemorrhagiae 16; COP, *L.* *interrogans* Icterohaemorrhagiae Copenhageni Winjberg; VER, *L.* *interrogans* Icterohaemorrhagiae Icterohaemorrhagiae Verdun; RGA, *L.* *interrogans* Icterohaemorrhagiae Icterohaemorrhagiae RGA; AKI, *L.* *interrogans* Autumnalis Autumnalis Akiyami; BIM, *L.* *kirschneri* Autumnalis Bim 1051; BAL, *L.* *borgpetersenii* Ballum Castellonis Castellon 3; CAN, *L.* *interrogans* Canicola Canicola Hond Utrecht IV; GRIP, *L.* *kirschneri* Grippotyphosa Grippotyphosa Moskva V; VAN, *L.* *kirschneri* Grippotyphosa Vanderhoedoni Kipod 179; HEB, *L.* *interrogans* Hebdomadis Kremastos Kremastos; PAN, *L.* *noguchii* Panama Panama CZ 214 K; POM, *L.* *interrogans* Pomona Pomona Pomona; MOZ, *L.* *kirschneri* Pomona Mozdok 5621; PYR, *L.* *interrogans* Pyrogenes Pyrogenes Salinem; SJ, *L.* *borgpetersenii* Sejroe Sejroe M 84; SAX, *L.* *interrogans* Sejroe Saxkoebing Mus 24; WOLF, *L.* *interrogans* Sejroe Wolffi 3705; HJ, *L.* *interrogans* Sejroe Hardjo Hardjoprajitno; TAR, *L.* *borgpetersenii* Tarassovi Tarassovi Perepelitsin; CYN, *L.* *kirschneri* Cynopteri Cynopteri 3522 C; MAN, *L*. noguchii Panama Mahnus TRVL/CAREC137774; JAV, *L.* *borgpetersenii* Javanica Javanica Veldrat Baravia 46; BAT, *L.* *interrogans* Bataviae Bataviae Van Tienen; MUN, *L.* *interrogans* Australis Muenchen Muenchen C90; AUS, *L.* *interrogans* Australis Australis Ballico; BRAT, *L.* *interrogans* Australis Bratislava Jez-Bratislava; T-, nuclease-free water.


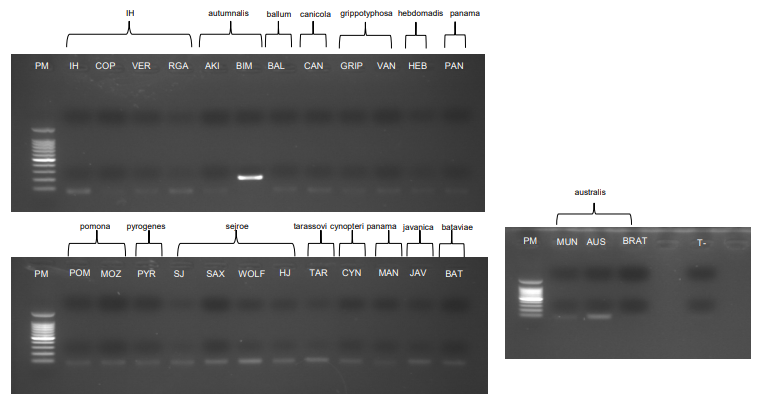


Supplementary Figure 2. Agarose gel images of PCR amplification with AUT_kir primers on 26 DNA samples (indicated in Table 3) extracted from strains of various serogroups to test efficiency and specificity. The row above the gel image depicts the serovar tested, while the bracket above indicates the according serogroup of *L.* spp.

PM, 100-pb ladder Invitrogen 15628019; IH, *L. interrogans* Icterohaemorrhagiae Icterohaemorrhagiae 16; COP, *L. interrogans* Icterohaemorrhagiae Copenhageni Winjberg; VER, *L. interrogans* Icterohaemorrhagiae Icterohaemorrhagiae Verdun; RGA, *L. interrogans* Icterohaemorrhagiae Icterohaemorrhagiae RGA; AKI, *L. interrogans* Autumnalis Autumnalis Akiyami; BIM, *L. kirschneri* Autumnalis Bim 1051; BAL, *L. borgpetersenii* Ballum Castellonis Castellon 3; CAN, *L. interrogans* Canicola Canicola Hond Utrecht IV; GRIP, *L. kirschneri* Grippotyphosa Grippotyphosa Moskva V; VAN, *L. kirschneri* Grippotyphosa Vanderhoedoni Kipod 179; HEB, *L. interrogans* Hebdomadis Kremastos Kremastos; PAN, *L. noguchii* Panama Panama CZ 214 K; POM, *L. interrogans* Pomona Pomona Pomona; MOZ, *L. kirschneri* Pomona Mozdok 5621; PYR, *L. interrogans* Pyrogenes Pyrogenes Salinem; SJ, *L. borgpetersenii* Sejroe Sejroe M 84; SAX, *L. interrogans* Sejroe Saxkoebing Mus 24; WOLF, *L. interrogans* Sejroe Wolffi 3705; HJ, *L. interrogans* Sejroe Hardjo Hardjoprajitno; TAR, *L. borgpetersenii* Tarassovi Tarassovi Perepelitsin; CYN, *L. kirschneri* Cynopteri Cynopteri 3522 C; MAN, *L. noguchii* Panama Mahnus TRVL/CAREC137774; JAV, *L. borgpetersenii* Javanica Javanica Veldrat Baravia 46; BAT, *L. interrogans* Bataviae Bataviae Van Tienen; MUN, *L. interrogans* Australis Muenchen Muenchen C90; AUS, *L. interrogans* Australis Australis Ballico; BRAT, *L. interrogans* Australis Bratislava Jez-Bratislava; T-, nuclease-free water.


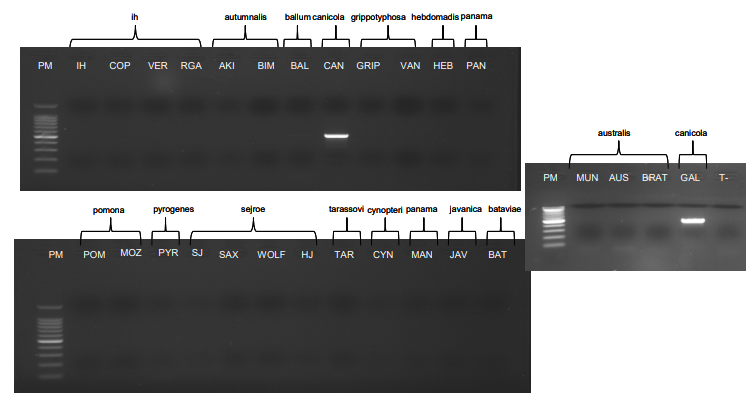


Supplementary Figure 3. Agarose gel images of PCR amplification with CAN primers on 26 DNA samples (indicated in Table 3) extracted from strains of various serogroups to test efficiency and specificity. The row above the gel image depicts the serovar tested, while the bracket above indicates the according serogroup of *L.* spp.

PM, 100-pb ladder Invitrogen 15628019; IH, *L. interrogans* Icterohaemorrhagiae Icterohaemorrhagiae 16; COP, *L. interrogans* Icterohaemorrhagiae Copenhageni Winjberg; VER, *L. interrogans* Icterohaemorrhagiae Icterohaemorrhagiae Verdun; RGA, *L. interrogans* Icterohaemorrhagiae Icterohaemorrhagiae RGA; AKI, *L. interrogans* Autumnalis Autumnalis Akiyami; BIM, *L. kirschneri* Autumnalis Bim 1051; BAL, *L. borgpetersenii* Ballum Castellonis Castellon 3; CAN, *L. interrogans* Canicola Canicola Hond Utrecht IV; GRIP, *L. kirschneri* Grippotyphosa Grippotyphosa Moskva V; VAN, *L. kirschneri* Grippotyphosa Vanderhoedoni Kipod 179; HEB, *L. interrogans* Hebdomadis Kremastos Kremastos; PAN, *L. noguchii* Panama Panama CZ 214 K; POM, *L. interrogans* Pomona Pomona Pomona; MOZ, *L. kirschneri* Pomona Mozdok 5621; PYR, *L. interrogans* Pyrogenes Pyrogenes Salinem; SJ, *L. borgpetersenii* Sejroe Sejroe M 84; SAX, *L. interrogans* Sejroe Saxkoebing Mus 24; WOLF, *L. interrogans* Sejroe Wolffi 3705; HJ, *L. interrogans* Sejroe Hardjo Hardjoprajitno; TAR, *L. borgpetersenii* Tarassovi Tarassovi Perepelitsin; CYN, *L. kirschneri* Cynopteri Cynopteri 3522 C; MAN, *L. noguchii* Panama Mahnus TRVL/CAREC137774; JAV, *L. borgpetersenii* Javanica Javanica Veldrat Baravia 46; BAT, *L. interrogans* Bataviae Bataviae Van Tienen; MUN, *L. interrogans* Australis Muenchen Muenchen C90; AUS, *L. interrogans* Australis Australis Ballico; BRAT, *L. interrogans* Australis Bratislava Jez-Bratislava; GAL, T-, nuclease-free water.


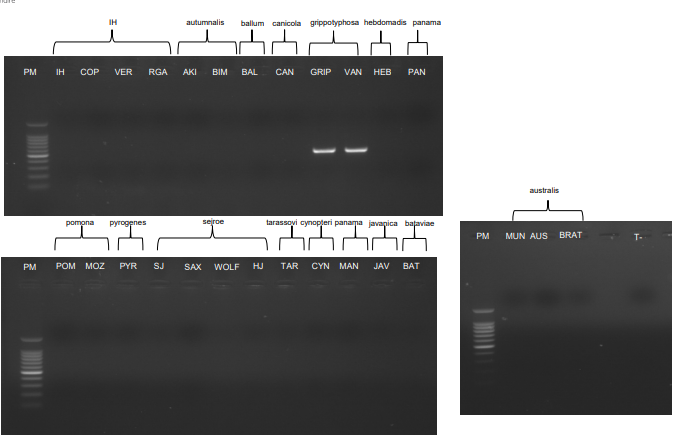


Supplementary Figure 4. Agarose gel images of PCR amplification with GRI primers on 26 DNA samples (indicated in Table 3) extracted from strains of various serogroups to test efficiency and specificity. The row above the gel image depicts the serovar tested, while the bracket above indicates the according serogroup of *L.* spp.

PM, 100-pb ladder Invitrogen 15628019; IH, *L. interrogans* Icterohaemorrhagiae Icterohaemorrhagiae 16; COP, *L. interrogans* Icterohaemorrhagiae Copenhageni Winjberg; VER, *L. interrogans* Icterohaemorrhagiae Icterohaemorrhagiae Verdun; RGA, *L. interrogans* Icterohaemorrhagiae Icterohaemorrhagiae RGA; AKI, *L. interrogans* Autumnalis Autumnalis Akiyami; BIM, *L. kirschneri* Autumnalis Bim 1051; BAL, *L. borgpetersenii* Ballum Castellonis Castellon 3; CAN, *L. interrogans* Canicola Canicola Hond Utrecht IV; GRIP, *L. kirschneri* Grippotyphosa Grippotyphosa Moskva V; VAN, *L. kirschneri* Grippotyphosa Vanderhoedoni Kipod 179; HEB, *L. interrogans* Hebdomadis Kremastos Kremastos; PAN, *L. noguchii* Panama Panama CZ 214 K; POM, *L. interrogans* Pomona Pomona Pomona; MOZ, *L. kirschneri* Pomona Mozdok 5621; PYR, *L. interrogans* Pyrogenes Pyrogenes Salinem; SJ, *L. borgpetersenii* Sejroe Sejroe M 84; SAX, *L. interrogans* Sejroe Saxkoebing Mus 24; WOLF, *L. interrogans* Sejroe Wolffi 3705; HJ, *L. interrogans* Sejroe Hardjo Hardjoprajitno; TAR, *L. borgpetersenii* Tarassovi Tarassovi Perepelitsin; CYN, *L. kirschneri* Cynopteri Cynopteri 3522 C; MAN, *L. noguchii* Panama Mahnus TRVL/CAREC137774; JAV, *L. borgpetersenii* Javanica Javanica Veldrat Baravia 46; BAT, *L. interrogans* Bataviae Bataviae Van Tienen; MUN, *L. interrogans* Australis Muenchen Muenchen C90; AUS, *L. interrogans* Australis Australis Ballico; BRAT, *L. interrogans* Australis Bratislava Jez-Bratislava; T-, nuclease-free water.


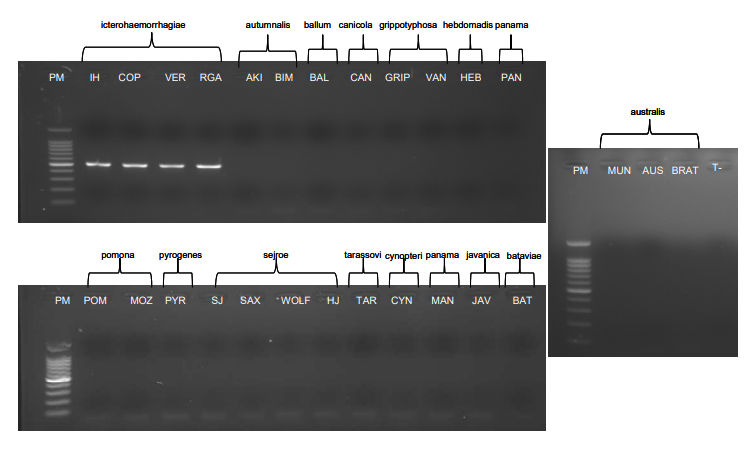


Supplementary Figure 5. Agarose gel images of PCR amplification with ICT primers on 26 DNA samples (indicated in Table 3) extracted from strains of various serogroups to test efficiency and specificity. The row above the gel image depicts the serovar tested, while the bracket above indicates the according serogroup of *L.* spp.

PM, 100-pb ladder Invitrogen 15628019; IH, *L. interrogans* Icterohaemorrhagiae Icterohaemorrhagiae 16; COP, *L. interrogans* Icterohaemorrhagiae Copenhageni Winjberg; VER, *L. interrogans* Icterohaemorrhagiae Icterohaemorrhagiae Verdun; RGA, *L. interrogans* Icterohaemorrhagiae Icterohaemorrhagiae RGA; AKI, *L. interrogans* Autumnalis Autumnalis Akiyami; BIM, *L. kirschneri* Autumnalis Bim 1051; BAL, *L. borgpetersenii* Ballum Castellonis Castellon 3; CAN, *L. interrogans* Canicola Canicola Hond Utrecht IV; GRIP, *L. kirschneri* Grippotyphosa Grippotyphosa Moskva V; VAN, *L. kirschneri* Grippotyphosa Vanderhoedoni Kipod 179; HEB, *L. interrogans* Hebdomadis Kremastos Kremastos; PAN, *L. noguchii* Panama Panama CZ 214 K; POM, *L. interrogans* Pomona Pomona Pomona; MOZ, *L. kirschneri* Pomona Mozdok 5621; PYR, *L. interrogans* Pyrogenes Pyrogenes Salinem; SJ, *L. borgpetersenii* Sejroe Sejroe M 84; SAX, *L. interrogans* Sejroe Saxkoebing Mus 24; WOLF, *L. interrogans* Sejroe Wolffi 3705; HJ, *L. interrogans* Sejroe Hardjo Hardjoprajitno; TAR, *L. borgpetersenii* Tarassovi Tarassovi Perepelitsin; CYN, *L. kirschneri* Cynopteri Cynopteri 3522 C; MAN, *L. noguchii* Panama Mahnus TRVL/CAREC137774; JAV, *L. borgpetersenii* Javanica Javanica Veldrat Baravia 46; BAT, *L. interrogans* Bataviae Bataviae Van Tienen; MUN, *L. interrogans* Australis Muenchen Muenchen C90; AUS, *L. interrogans* Australis Australis Ballico; BRAT, *L. interrogans* Australis Bratislava Jez-Bratislava; T-, nuclease-free water.


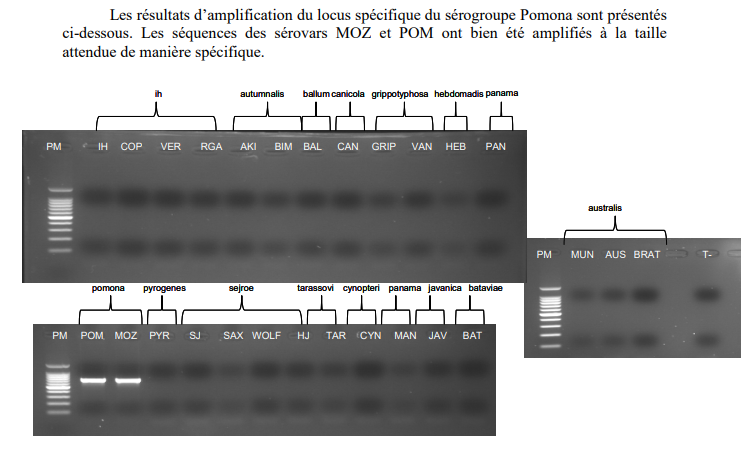


Supplementary Figure 6. Agarose gel images of PCR amplification with POM-2 primers on 26 DNA samples (indicated in Table 3) extracted from strains of various serogroups to test efficiency and specificity. The row above the gel image depicts the serovar tested, while the bracket above indicates the according serogroup of *L.* spp.

PM, 100-pb ladder Invitrogen 15628019; IH, *L. interrogans* Icterohaemorrhagiae Icterohaemorrhagiae 16; COP, *L. interrogans* Icterohaemorrhagiae Copenhageni Winjberg; VER, *L. interrogans* Icterohaemorrhagiae Icterohaemorrhagiae Verdun; RGA, *L. interrogans* Icterohaemorrhagiae Icterohaemorrhagiae RGA; AKI, *L. interrogans* Autumnalis Autumnalis Akiyami; BIM, *L. kirschneri* Autumnalis Bim 1051; BAL, *L. borgpetersenii* Ballum Castellonis Castellon 3; CAN, *L. interrogans* Canicola Canicola Hond Utrecht IV; GRIP, *L. kirschneri* Grippotyphosa Grippotyphosa Moskva V; VAN, *L. kirschneri* Grippotyphosa Vanderhoedoni Kipod 179; HEB, *L. interrogans* Hebdomadis Kremastos Kremastos; PAN, *L. noguchii* Panama Panama CZ 214 K; POM, *L. interrogans* Pomona Pomona Pomona; MOZ, *L. kirschneri* Pomona Mozdok 5621; PYR, *L. interrogans* Pyrogenes Pyrogenes Salinem; SJ, *L. borgpetersenii* Sejroe Sejroe M 84; SAX, *L. interrogans* Sejroe Saxkoebing Mus 24; WOLF, *L. interrogans* Sejroe Wolffi 3705; HJ, *L. interrogans* Sejroe Hardjo Hardjoprajitno; TAR, *L. borgpetersenii* Tarassovi Tarassovi Perepelitsin; CYN, *L. kirschneri* Cynopteri Cynopteri 3522 C; MAN, *L. noguchii* Panama Mahnus TRVL/CAREC137774; JAV, *L. borgpetersenii* Javanica Javanica Veldrat Baravia 46; BAT, *L. interrogans* Bataviae Bataviae Van Tienen; MUN, *L. interrogans* Australis Muenchen Muenchen C90; AUS, *L. interrogans* Australis Australis Ballico; BRAT, *L. interrogans* Australis Bratislava Jez-Bratislava; T-, nuclease-free water.


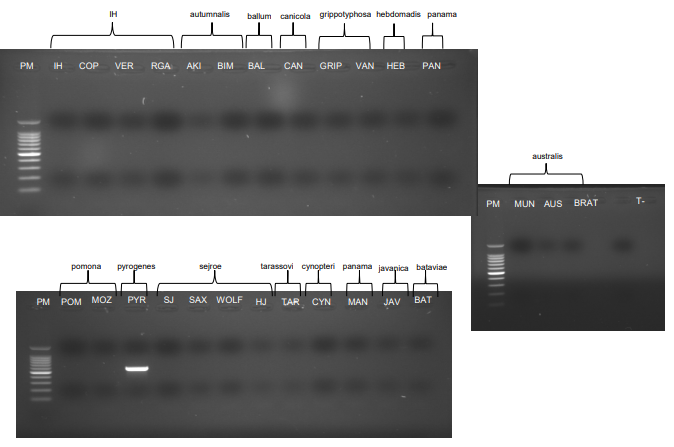


Figure 7. Agarose gel images of PCR amplification with PYR-2 primers on 26 DNA samples (indicated in Table 3) extracted from strains of various serogroups to test efficiency and specificity. The row above the gel image depicts the serovar tested, while the bracket above indicates the according serogroup of *L.* spp.

PM, 100-pb ladder Invitrogen 15628019; IH, *L. interrogans* Icterohaemorrhagiae Icterohaemorrhagiae 16; COP, *L. interrogans* Icterohaemorrhagiae Copenhageni Winjberg; VER, *L. interrogans* Icterohaemorrhagiae Icterohaemorrhagiae Verdun; RGA, *L. interrogans* Icterohaemorrhagiae Icterohaemorrhagiae RGA; AKI, *L. interrogans* Autumnalis Autumnalis Akiyami; BIM, *L. kirschneri* Autumnalis Bim 1051; BAL, *L. borgpetersenii* Ballum Castellonis Castellon 3; CAN, *L. interrogans* Canicola Canicola Hond Utrecht IV; GRIP, *L. kirschneri* Grippotyphosa Grippotyphosa Moskva V; VAN, *L. kirschneri* Grippotyphosa Vanderhoedoni Kipod 179; HEB, *L. interrogans* Hebdomadis Kremastos Kremastos; PAN, *L. noguchii* Panama Panama CZ 214 K; POM, *L. interrogans* Pomona Pomona Pomona; MOZ, *L. kirschneri* Pomona Mozdok 5621; PYR, *L. interrogans* Pyrogenes Pyrogenes Salinem; SJ, *L. borgpetersenii* Sejroe Sejroe M 84; SAX, *L. interrogans* Sejroe Saxkoebing Mus 24; WOLF, *L. interrogans* Sejroe Wolffi 3705; HJ, *L. interrogans* Sejroe Hardjo Hardjoprajitno; TAR, *L. borgpetersenii* Tarassovi Tarassovi Perepelitsin; CYN, *L. kirschneri* Cynopteri Cynopteri 3522 C; MAN, *L. noguchii* Panama Mahnus TRVL/CAREC137774; JAV, *L. borgpetersenii* Javanica Javanica Veldrat Baravia 46; BAT, *L. interrogans* Bataviae Bataviae Van Tienen; MUN, *L. interrogans* Australis Muenchen Muenchen C90; AUS, *L. interrogans* Australis Australis Ballico; BRAT, *L. interrogans* Australis Bratislava Jez-Bratislava; T-, nuclease free-water.


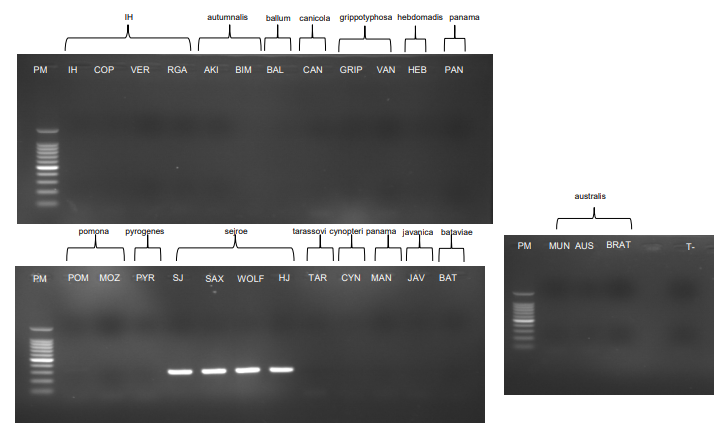


Supplementary Figure 8. Agarose gel images of PCR amplification with SEJ primers on 26 DNA samples (indicated in Table 3) extracted from strains of various serogroups to test efficiency and specificity. The row above the gel image depicts the serovar tested, while the bracket above indicates the according serogroup of *L.* spp.

PM, 100-pb ladder Invitrogen 15628019; IH, *L. interrogans* Icterohaemorrhagiae Icterohaemorrhagiae 16; COP, *L. interrogans* Icterohaemorrhagiae Copenhageni Winjberg; VER, *L. interrogans* Icterohaemorrhagiae Icterohaemorrhagiae Verdun; RGA, *L. interrogans* Icterohaemorrhagiae Icterohaemorrhagiae RGA; AKI, *L. interrogans* Autumnalis Autumnalis Akiyami; BIM, *L. kirschneri* Autumnalis Bim 1051; BAL, *L. borgpetersenii* Ballum Castellonis Castellon 3; CAN, *L. interrogans* Canicola Canicola Hond Utrecht IV; GRIP, *L. kirschneri* Grippotyphosa Grippotyphosa Moskva V; VAN, *L. kirschneri* Grippotyphosa Vanderhoedoni Kipod 179; HEB, *L. interrogans* Hebdomadis Kremastos Kremastos; PAN, *L. noguchii* Panama Panama CZ 214 K; POM, *L. interrogans* Pomona Pomona Pomona; MOZ, *L. kirschneri* Pomona Mozdok 5621; PYR, *L. interrogans* Pyrogenes Pyrogenes Salinem; SJ, *L. borgpetersenii* Sejroe Sejroe M 84; SAX, *L. interrogans* Sejroe Saxkoebing Mus 24; WOLF, *L. interrogans* Sejroe Wolffi 3705; HJ, *L. interrogans* Sejroe Hardjo Hardjoprajitno; TAR, *L. borgpetersenii* Tarassovi Tarassovi Perepelitsin; CYN, *L. kirschneri* Cynopteri Cynopteri 3522 C; MAN, *L. noguchii* Panama Mahnus TRVL/CAREC137774; JAV, *L. borgpetersenii* Javanica Javanica Veldrat Baravia 46; BAT, *L. interrogans* Bataviae Bataviae Van Tienen; MUN, *L. interrogans* Australis Muenchen Muenchen C90; AUS, *L. interrogans* Australis Australis Ballico; BRAT, *L. interrogans* Australis Bratislava Jez-Bratislava; T-, nuclease-free water.
